# Air Pollution and Lung Cancer: Contributions of Extracellular Vesicles as Pathogenic Mechanisms and Clinical Utility

**DOI:** 10.1007/s40572-023-00421-8

**Published:** 2023-12-06

**Authors:** Jonathan González-Ruíz, Andrea A.Baccarelli, David Cantu-de-Leon, Diddier Prada

**Affiliations:** 1 Instituto Nacional de Cancerología, 14080 Mexico City, Mexico; 2 Mailman School of Public Health, Department of Environmental Health Sciences, Columbia University, New York City, NY 10032 USA; 3 Department of Population Health Science and Policy and the Department of Environmental Medicine and Public Health, Institute for Health Equity Research, Icahn School of Medicine at Mount Sinai, 1 Gustave L. Levy Pl · (212) 241-6500, Room L2-38, New York City, NY 10029 USA

**Keywords:** Air pollution, Lung cancer, Extracellular vesicles, Biomarkers, Therapeutic targets

## Abstract

**Purpose of Review:**

This review addresses the pressing issue of air pollution’s threat to human health, focusing on its connection to non-small cell lung cancer (NSCLC) development. The aim is to explore the role of extracellular vesicles (EVs) as potential pathogenic mechanisms in lung cancer, including NSCLC, induced by air pollutants.

**Recent Findings:**

Recent research highlights EVs as vital mediators of intercellular communication and key contributors to cancer progression. Notably, this review emphasizes the cargo of EVs released by both cancerous and non-cancerous lung cells, shedding light on their potential role in promoting various aspects of tumor development.

**Summary:**

The review underscores the importance of comprehending the intricate interplay between air pollution, biological damage mechanisms, and EV-mediated communication during NSCLC development. Major takeaways emphasize the significance of this understanding in addressing air pollution-related lung cancer. Future research avenues are also highlighted, aiming to enhance the applicability of EVs for diagnosis and targeted therapies, ultimately mitigating the inevitable impact of air pollution on NSCLC development and treatment.

## Introduction

In recent years, the adverse effects of air pollution on human health have become a growing concern worldwide [1, 2]. The quality of the air we breathe plays a crucial role in maintaining our well-being, particularly when it comes to lung health [3]. Prolonged exposure to air pollutants may induce a wide range of respiratory symptoms, including coughing, wheezing, shortness of breath, and chest tightness, all of them linked with acute and long-term effects [4]. Air pollutants, consisting of a complex mixture of harmful particles and gases, have been linked to a wide range of respiratory disorders, including asthma, chronic obstructive pulmonary disease (COPD), reduced lung function, and lung cancer, in particular, non-small cell lung cancer (NSCLC) [5]. The association between environmental air pollutants and lung cancer has been extensively studied [3]. In October 2013, the specialized cancer agency of the World Health Organization, the International Agency for Research on Cancer (IARC) announced that outdoor air pollution was classified as carcinogenic to humans (Group 1) [6]. Further epidemiological studies have consistently demonstrated a strong link between prolonged exposure to air pollutants and the development of lung cancer [7].

Lung cancer ranks as the most prevalent cancer and remains the primary cause of cancer-related deaths and is a significant global health issue, with current worldwide statistics reflecting its magnitude. Across the globe, lung cancer stands as the most common cancer, and its impact is substantial. In 2022, there were over 2 million new cases of lung cancer reported, accounting for approximately 11% of all new cancer diagnoses [8]. Unfortunately, lung cancer continues to be the leading cause of cancer-related deaths globally. While this cancer affects both men and women, the statistics reveal significant sex disparities. Among men, lung cancer remains the leading cause of cancer-related mortality. Among women, it ranks second only to breast cancer, being a major cause of mortality among them. These statistics underscore the importance of understanding both the overall prevalence of lung cancer and the gender-specific differences in its incidence and consequences [9]. It is also the third most common cancer, following breast and colorectal cancers, and the second leading cause of cancer death after breast cancer among women vulnerable groups around the world include individuals with limited access to healthcare, low socioeconomic status, heavy tobacco user, and those living in highly polluted areas [10].

Air pollution may activate several cellular, molecular, and systemic changes, including inflammation, oxidative damage, microthrombosis, epigenomic changes, and activation of several other cellular responses, including the release of extracellular vesicles (EVs) [11••]. EVs encompass a heterogeneous group of vesicles that can be classified into three main subtypes, microvesicles, exosomes, and apoptotic bodies [12]. EVs are membrane-bound structures that are shed from the plasma membrane of cells [13]. They encapsulate a diverse range of molecules, including peptides, nucleic acids (such as microRNAs, mRNAs, and long noncoding RNAs), lipids, and metabolites. This cargo can be transferred to recipient cells, modulating their function and behavior [14]. In normal cells, EVs play an important role in intercellular communication by allowing cells to exchange information and signals with each other [15]. EVs have been shown to be involved in a variety of physiological and pathological processes, including immune regulation, tissue repair and regeneration, inflammation, and angiogenesis [16]. EVs can be released practically by any cell, including cancer cells [17], and have been implicated in tumor growth, metastasis, and drug resistance [18]. This review examines the mechanisms of air pollution’s impact on lung cancer development, with a focus on the contribution of extracellular vesicles to carcinogenesis and cancer progression. Additionally, it explores the potential utility of these vesicles in clinical settings for lung cancer (i.e., NSCLC).

## Air Pollution and Its Impact on Human Health

Air pollution components contain particulate matter (PM) and gases, including volatile organic compounds. Particulate matter is a common component of air pollution and consists of tiny particles suspended in the air [19]. These particles can be classified based on their size, with particles with a diameter of 2.5 µm or less (PM_2.5_) and PM_10_ (10 µm or less), also called coarse particles, and ultrafine particles (PM_0.1_), all of them being studied in relation to lung cancer [20]. PM_2.5_ can penetrate deep into the respiratory system, reaching the lungs’ alveolar regions [21]. Inhaled fine PM deposited on the surface of the airways may either stay intact or partially dissolve but can also be cleared by mucociliary clearance and phagocytosis [22]. PM can carry various carcinogens, such as polycyclic aromatic hydrocarbons (PAHs), heavy metals, and organic compounds, which have been linked to cancer development, including in the lungs [23]. Air pollution gases include nitrogen oxides (NOx: NO and NO_2_) and sulfur dioxide (SO_2_), which are produced primarily from combustion processes, such as those occurring in vehicle engines, power plants, and industrial facilities [24]. These pollutants can react with other compounds in the atmosphere to form secondary pollutants, such as nitric acid (HNO_3_) and ozone (O_3_) [25]. Studies have shown that exposure to nitrogen oxides, particularly in combination with other pollutants, increases the risk of lung cancer [26]. O_3_, a key component of photochemical smog, is another important air pollutant formed by the reaction of nitrogen oxides with volatile organic compounds (VOCs) in the presence of sunlight [27]. Prolonged exposure to ozone has been associated with adverse respiratory effects, and recent research also indicates a potential link between ozone exposure and lung cancer development [28]. Volatile organic compounds are also present in air pollution and are emitted from a wide range of sources, including industrial processes, vehicle emissions, and solvents [29]. Some VOCs, such as benzene, formaldehyde, and 1,3-butadiene, have been classified as carcinogens by the IARC [30]. All these pollutants may act in cells and tissues individually but also as mixtures. Therefore, air pollution includes a variety of compounds that, after long-term exposure, can contribute to an increased risk of developing lung cancer, particularly non-small cell lung cancer (NSCLC) [31].

## Air Pollution and the Development of Lung Cancer: Potential Mechanisms of Damage and Relevance of EVs

**Fig. 1:**
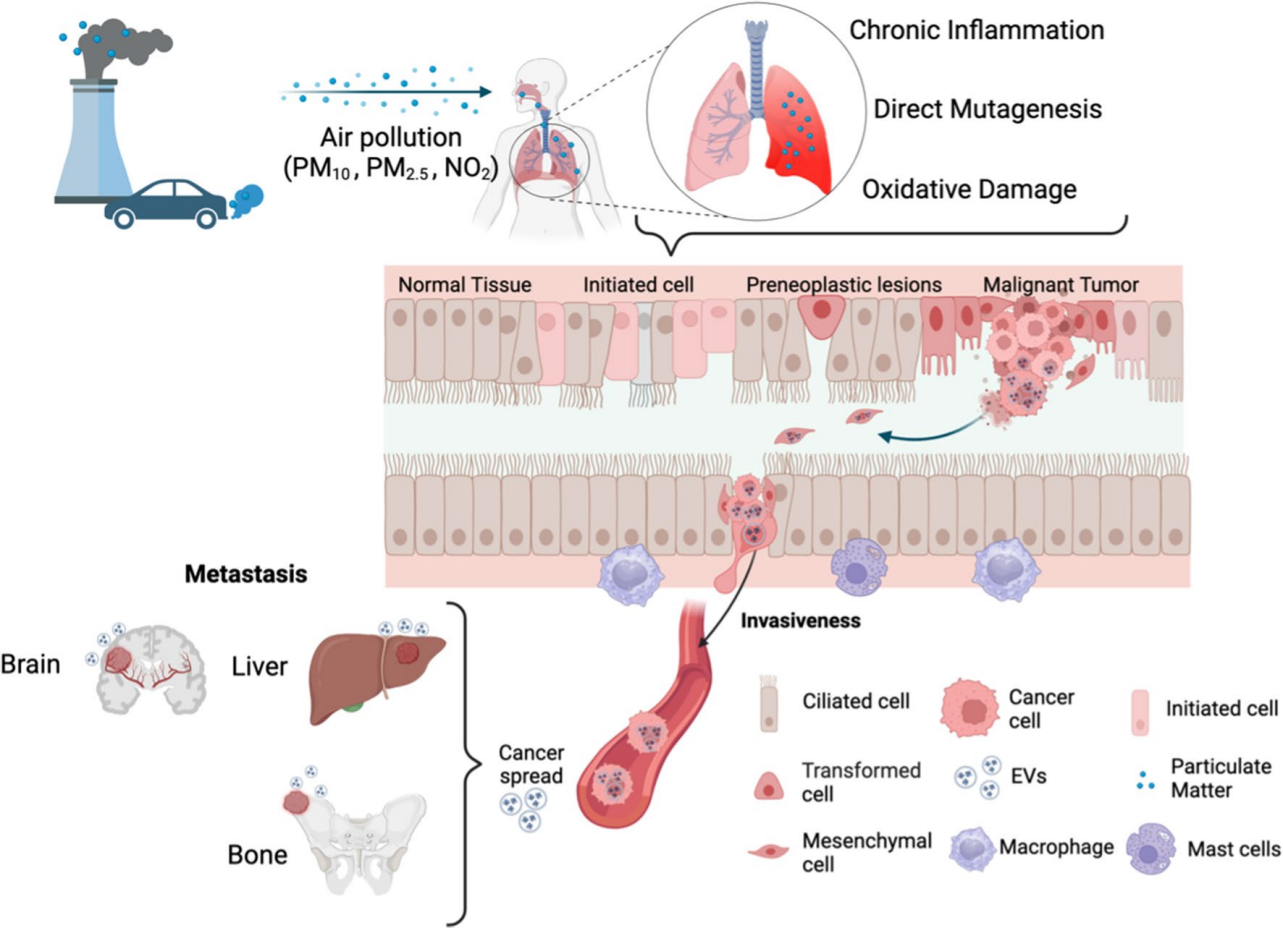
Air pollution–related lung cancer and role of extracellular vesicles

Several mechanisms that could lead to lung carcinogenesis are activated by air pollutants, individually and as mixtures. The most studied factors contributing to lung carcinogenesis include low-grade, chronic inflammation, oxidative stress, direct mutagenesis, epigenetic changes, and mitochondrial and endothelial dysfunction, but there are many others. EVs can contribute to some of these mechanisms to carry on signals to other cells and even contribute to adapting to air pollution damage. A summary of air pollution-related damage and the role of EVs is shown in Fig. 1.

### Air Pollution, Low-Grade Chronic Inflammation, and Lung Cancer

Chronic low-grade inflammation, characterized by persistent mild immune activation, has garnered significant attention in the realm of environmentally related carcinogenesis. This subtle yet prolonged immune response is recognized as a fundamental factor in cancer development, notably playing a pivotal role in the promotion and progression of various cancer types, including air pollution-induced lung cancer [32]. Prolonged exposure to environmental factors, such as carcinogenic air pollutants, is frequently associated with the initiation of low-grade inflammation. For example, Darras et al. (2022), evaluating 3074 individuals, revealed that short-term exposure to air pollution is linked to elevated serum levels of high-sensitivity C-reactive protein (hsCRP) in adult residents of urban areas [33]. On the other hand, Kim et al. (2020) delved into the effects of long-term exposure to ambient air pollution on high-sensitivity, low-grade inflammation in 60,581 participants. The research identified elevated hs-CRP as a marker of low-grade inflammation associated with exposure to PM_10_, PM_2.5_, and SO_2_[34]. These findings underscore the potential role of chronic, low-grade inflammation in explaining the adverse health effects of air pollution [34].

In contrast, high-grade inflammation typically results from acute and severe exposures, such as intense infections or trauma. While it exerts an immediate and robust impact on the immune system, it is usually of a transient nature and not a sustained state [35]. In the context of cancer, high-grade inflammation can lead to tissue damage and the creation of a pro-inflammatory environment [36]. However, its transient nature distinguishes it from chronic low-grade inflammation [37]. The latter, due to its persistence, is widely recognized for its role in cancer development and progression [38], warranting intensive research to comprehend its implications in carcinogenesis, particularly in diseases like NSCLC induced by environmental pollutants.

Chronic low-grade inflammation is a well-known contributor to carcinogenesis [32]. Air pollution–related inflammation involves the release of numerous mediators, including cytokines, chemokines, and growth factors, which act as messengers to orchestrate the immune response that usually occurs in low-grade and chronic settings. [35] These mediators are produced by immune cells, such as macrophages, neutrophils, and lymphocytes, as well as by stromal cells [39]. Air pollution–related inflammation contributes to DNA damage, activation of oncogenic pathways, inhibition of apoptosis, recruitment of immune cells, and angiogenesis, among others. EVs have been suggested to play critical roles in both exacerbating and mitigating chronic inflammation through cell-to-cell interactions, as EVs transport cargoes that modulate cell signaling and injury responses, exacerbating the inflammatory reactions over time [35]. Air pollution–related inflammation can also cause reactive oxygen species (ROS) and reactive nitrogen species (RNS) release [40•].

### Air Pollution, Oxidative Stress, and Lung Cancer

Air pollution is a complex mixture of harmful substances and particles present in the atmosphere, arising from various sources like industrial emissions, vehicle exhaust, and more. This contamination has been a growing concern for public health due to its association with oxidative stress, a pivotal factor in the development and progression of lung cancer. The most significant environmental pollutants include PMs, VOCs, O_3_, and PAHs. While PAHs are consistently bound to PMs, they have a substantial impact on oxidative damage within the lungs because they are closely associated with it [41•].

Oxidative stress involves the imbalance between the production of ROS and the capacity of antioxidant systems to neutralize them [42]. ROS are unstable and highly reactive molecules that can cause damage to cells by interacting with DNA, lipids, and proteins [43]. These highly reactive molecules can cause oxidative stress and direct DNA damage. This chronic oxidative damage/modification process contributes to several tissue dysfunction and disease over time [44].

NADPH oxidases (NOX) are primary ROS-generating enzymes [45]. Activation of the NOX-2 enzyme leads to the formation of the superoxide radical (O2^•−^) that can either react with NO to create harmful peroxynitrite or undergo modification through superoxide dismutase (SOD) which converts O2^•−^ into harmful hydrogen peroxide[46]. This hydrogen peroxide, in the presence of ferrous iron, can be converted into hydroxyl radicals (•OH) through the Fenton reaction [47]. •OH can directly attack DNA molecules, and •OH reacts with the various hydrogen atoms of the deoxyribose, leading to damage that can be transient or not [48]. One significant type of DNA damage caused by •OH is the formation of 8-oxoguanine lesions [49]. 8-Oxoguanine is a modified DNA base resulting from the oxidation of guanine, one of the four DNA bases.

Additionally, a critical aspect of this oxidative stress is the role of cytochrome P450-dependent monooxygenases (CYPs) and peroxidases in the metabolism of PAHs [50]. There are two primary pathways for PAH activation. Initially, CYP-mediated monooxygenation leads to the formation of highly reactive epoxides, which subsequently hydrolyze into dihydrodiols (“diols”) and diol-epoxides. Furthermore, PAHs can be converted into ortho-quinones, resulting in the generation of reactive oxygen species (ROS) through redox cycling. Additionally, the one-electron oxidation of specific carcinogenic PAHs, catalyzed by CYPs or peroxidases, produces radical cations with the potential to induce DNA damage [51]. This intricate interplay between PAH metabolism and the formation of DNA-damaging species underscores their possible role in carcinogenesis [52].

Oxidative stress is also known to trigger the release of EVs as a cellular response to restore the redox balance [53]. Interactions between stressed immune cells and lung epithelial cells via EVs also contribute to the development of lung inflammation [54]. When lung cells undergo stress, infection, or hyperoxia, they release EVs carrying harmful substances into the airway surface liquid, disrupting the balanced communication between lung epithelium and immune cells [55]. This disruption leads to pulmonary inflammation and tissue injury.

### Activation of Oncogenic Pathways by Air Pollutants

Air pollution–related pro-inflammatory mediators, such as tumor necrosis factor-alpha (TNF-α), interleukin-6 (IL-6), and interleukin-1β (IL-1β), create pro-inflammatory environments that promote tumor development [56]. Inflammatory signaling pathways, such as nuclear factor-kappa B (NF-κB) and signal transducer and activator of transcription 3 (STAT3), are frequently activated in lung cancer [57]. These pathways regulate the expression of genes involved in cell survival, proliferation, angiogenesis, and metastasis, providing a favorable environment for tumor growth [58]. Inflammation creates a tumor-promoting microenvironment by influencing various components of the tumor microenvironment, including immune cells, fibroblasts, endothelial cells, and extracellular matrix [59]. Inflammatory mediators can recruit and activate immune cells, such as tumor-associated macrophages (TAMs) and myeloid-derived suppressor cells, which can secrete factors that support tumor growth, angiogenesis, and immune evasion [60, 61]. TAMs can polarize to an M2 phenotype (i.e., alternatively activated macrophages), which promotes tumor progression and suppresses anti-tumor immune responses [62]. Inflammation also stimulates the production of growth factors and cytokines that promote angiogenesis, the formation of new blood vessels essential for tumor nourishment [63]. Furthermore, inflammatory cells and stromal cells contribute to the remodeling of the extracellular matrix, facilitating tumor invasion and metastasis. Inflammation plays a multifaceted role in lung cancer development, influencing various aspects of tumor initiation, growth, and progression [64]. Therefore, chronic inflammation induces DNA damage, creates a tumor-promoting microenvironment, impairs immune surveillance, and promotes lung angiogenesis, facilitating lung carcinogenesis [65]. One of the concerning aspects of air pollution is its potential to induce mutagenesis, leading to genetic alterations in living organisms [66]. Laboratory mice located near polluted industrial areas (1 km downwind from two integrated steel mills) showed higher heritable mutation frequency at tandem-repeat DNA loci compared with those at a reference site 30 km away, and this damage was due primarily to an increase in mutations inherited through the paternal germline [66]. Mutations in key regulatory genes can disrupt cellular processes, leading to uncontrolled cell growth and the development of cancer [67]. These results strongly suggest that mitigation of air pollution is crucial to reduce its mutagenic potential on lung cells.

### Air Pollution-Induced Damage and EVs

Air pollutants have been linked to an augmented secretion of EVs. EVs derived from pulmonary artery endothelial cells (PAECs) may promote over-proliferation and apoptosis resistance of pulmonary artery smooth muscle cells (PASMC), contributing to pulmonary vascular remodeling and hypertension [68]. These EVs harbor microRNAs, including miR-17, miR-20a, miR-21, and miR-145, which modulate the expression of genes like BMPR2 and pro-inflammatory factors [69], potentially influencing specific signaling pathways in recipient cells. EVs also play a role in the development of the tumor microenvironment [70•]. Once transformed, cancer cells can release EVs that contain pro-inflammatory factors, such IL-6 and TNF-α, which can activate signaling pathways in stromal cells and promote their migration to the tumor site [71]. EVs can also induce angiogenesis by transferring angiogenic factors, such as vascular endothelial growth factor (VEGF), to endothelial cells [72]. Air pollution-derived DNA damage and its physiological response can activate cellular processes that promote the release of EVs containing damaged DNA, oncogenic cargo, or genetic material with altered expression profiles, thereby contributing to lung cancer development [53]. Air pollution have been shown to induce epigenetic changes in lung cells [73], including DNA methylation, histone modifications, and alterations in noncoding RNA expression, which may also influence extracellular vesicles biogenesis and changes in cargo content, potentially promoting lung cancer initiation and progression [74]. Air pollution–mediated cell communication disruption [75], through direct effects on cellular membrane integrity, intercellular junctions, or signaling pathways, may also lead to the release of EVs as a compensatory mechanism [76]. The released EVs can then mediate aberrant cell signaling, contribute to cellular transformation, and facilitate tumor growth [77].

The contents of EVs have pleiotropic effects, including the oncogenic cargo, may remain fully functional. This cargo can induce alterations in the cellular microenvironment. Essentially, cells damaged by air pollutants may release EVs containing various factors, such as growth factors, cytokines, and angiogenic factors. These components can disrupt cellular homeostasis, promote cell proliferation by altering the microenvironment, and stimulate angiogenesis. Additionally, they hold the potential to transform recipient cells [78]. EVs released in response to air pollution-induced damage can facilitate the dissemination of carcinogenic compounds and toxic substances to distant organs, contributing to the metastatic spread of lung cancer [79]. EVs pro-inflammatory factors, growth factors, and angiogenic factors establish a tumor-promoting microenvironment characterized by chronic inflammation, increased cell proliferation, and the formation of new blood vessels [80]. EVs may carry vascular VEGF, promoting angiogenesis and the formation of new blood vessels that supply nutrients and oxygen to the growing tumor [81]. Moreover, EVs can transfer pro-inflammatory factors, such as IL-6 and TNF-α, promoting an inflammatory milieu that supports tumor growth [82]. EVs can also modulate gene expression in recipient cells by transferring microRNAs (miRNAs) and other small RNAs [83]. For example, cancer cells can release EVs containing miRNAs that inhibit the expression of tumor suppressor genes in recipient cells [84]. EVs derived from damaged lung cells can transfer “oncogenic cargo,” such as mutated genes and proteins, to recipient cells, promoting malignant transformation and uncontrolled cell growth [85]. EVs have been implicated in several aspects of carcinogenesis, particularly tumor growth [70•] which is promoted tumor by inducing angiogenesis and altering the tumor microenvironment [14]. EVs can transfer oncogenic proteins and activate signaling pathways that promote cell proliferation, invasion, and angiogenesis [84]. EVs can also enhance potential metastasis by modulating the behavior of transformed cells and promoting their migration and invasion [86].

EVs also play a crucial role in tumor invasion by influencing cancer cell behavior. They can transfer proteins and genetic material that induce changes in recipient cells, leading to enhanced migration and invasion capabilities [87•]. For instance, EVs can transfer oncogenic proteins that activate signaling pathways involved in cell motility and invasion [88]. Additionally, they can transfer miRNAs that suppress the expression of metastasis suppressor genes, facilitating the invasive potential of cancer cells [80].

EVs are implicated in the process of metastasis, the spread of cancer cells to distant sites in the body [88]. EVs can prepare the pre-metastatic niche by modifying the microenvironment of future metastatic sites [85]. They can promote the recruitment and activation of immune cells, release factors that modify the extracellular matrix, and induce angiogenesis at these sites, thus creating a favorable environment for cancer cell survival and colonization [85]. EVs have been shown to mediate the communication between primary tumor cells and distant organs to facilitate the establishment of metastases [89]. EVs can be released into the bloodstream and carry cargo that prepares distant sites for the arrival of metastatic cells [88]. They can prime target cells in these organs by altering gene expression, inducing phenotypic changes, and promoting the formation of a supportive microenvironment [90]. Once the carcinogenic process has occurred, EVs can transfer drug-resistance genes and pump drugs out of cells, leading to the development of multidrug resistance in cancer cells [91]. Cancer cells can release EVs that contain drug efflux pumps, which can pump drugs out of cells and reduce their efficacy [92]. Additionally, EVs can transfer drug resistance genes from one cell to another, leading to the development of multidrug resistance [93]. EVs may also play a significant role in cancer spread, facilitating the dissemination of cancer cells from the primary tumor to distant sites [94]. While the precise mechanisms underlying their involvement in lung cancer dissemination are still being elucidated, the potential impact of air pollution on EVs-mediated processes is a topic of increasing interest [77, 95].

Investigating the interplay between EVs and air pollution in lung cancer may provide valuable insights into the mechanisms driving malignant transformation and open new avenues for the development of preventive and early therapeutic strategies for lung cancer prevention [96]. For example, targeting EV biogenesis, release, or uptake by recipient cells could potentially disrupt their pro-tumorigenic effects [97]. Additionally, profiling the cargo of EVs may offer valuable early diagnostic information, enabling the development of personalized preventive strategies [98].

## The Importance of EVs in the Clinical Context of Lung Cancer

EVs have emerged as key players in the pathophysiology of various diseases, including lung cancer [99]. In this context, EVs may have immense clinical impact, serving as potential diagnostic biomarkers, therapeutic tools, and modulators of tumor progression and drug resistance [100]. EVs can be easily isolated from various biofluids, including blood, sputum, urine, and bronchoalveolar lavage fluid, allowing for convenient and repeatable sampling; therefore, they are highly promising for lung cancer early detection, diagnosis, prognosis, and treatment response biomarkers [101]. EVs derived from lung cancer cells, or the tumor microenvironment carry specific signatures (e.g., miRNAs, metabolites, peptides) that reflect inside the cellular state, making them potential easy-to-access biomarkers for lung cancer [102]. Liquid biopsies, which involve the analysis of circulating tumor-derived components, including EVs, have emerged as a promising approach for lung cancer [103]. The analysis of EV cargo, including specific proteins, nucleic acids, and mutations, can provide valuable information about tumor heterogeneity, genetic alterations, and treatment resistance [98]. For example, EV-miRNAs have shown diagnostic potential in lung cancer. EV-miRNAs such as miRNA-21, miRNA-155, miRNA-205, miRNA-19a, miRNA-19b, miRNA-30b, and miRNA-20a have been considered potential biomarkers for diagnosing lung cancer [104, 105]. Other miRNA profiles in EVs have been associated with disease progression, metastasis, and response to therapy [101]. These miRNAs can serve as biomarkers to stratify patients, predict prognosis, and monitor treatment response [106]. Surface proteins displayed on EVs can also serve as diagnostic markers in lung cancer [107].

For example, lung cancer-specific surface markers, such as Complement factor H-related protein 4 (CFHR4) and Coagulation factor XIII A chain (F13A1). Both proteins have been described as exclusively deregulated in lung cancer patients when compared to healthy donors[108]. Although their functions in other types of cancer remain unclear, Pedersen et al. proposed these two proteins as specific markers for lung cancer. Notably, both proteins were found to be localized within exosomes [108]. Although lung carcinogenesis may have an important contribution from air pollutants, we do not currently have tools to differentiate lung cancer induced by air pollution or by other causes. So, once the malignant tumor exists, it is considered a unique entity (i.e., NSCLC). In the next lines, we will describe the potential utility of EVs for this entity, not specifically for air pollution-induced lung cancer.

### EVs in Lung Cancer Diagnosis

Very few studies have tried to address the question of whether EVs could be relevant for lung cancer diagnosis. However, Zhang et al. identified 302 differentially secreted proteins from exosome-induced lung fibroblasts and verified that the proteins secreted by exosome-activated fibroblasts could result in alterations of extracellular matrix components and promote the growth of cancer cells [109]. Furthermore, based on the genes developed from differentially secreted proteins induced by EVs, they attempted to identify a potential diagnostic marker and prognostic signature for NSCLC, which may be useful in clinical practice [109]. Novikova et al., by applying targeted mass spectrometry with stable isotope-labeled peptide standards, assessed the levels of 28 EV-associated proteins in vesicles derived from the lung cancer cell lines NCI-H23 and A549, but also in plasma samples from 34 lung cancer patients and 23 healthy volunteers. They found that the most diagnostically potent markers were Talin-1 (TLN1), tubulin alpha 4a (TUBA4A), and heat shock protein family A (Hsp70) member 8 (HSPA8) and that the obtained EV proteomic signature allowed them to distinguish between the lung adenocarcinoma and squamous cell carcinoma histological types [110]. Based on these findings, it is possible that an EV biomarker will soon be available as a tool for lung cancer diagnosis using peripheral blood, saliva, or bronchoalveolar samples.

### EVs in Lung Cancer Progression and Metastasis

As we mentioned in the first part of this review, EVs play a crucial role in the complex processes of tumor progression and metastasis [79]. EVs can modulate the tumor microenvironment, promote angiogenesis, enhance tumor cell survival and proliferation, facilitate immune evasion, and promote epithelial-mesenchymal transition (EMT) [111]. Tumor-derived EVs can modulate the surrounding stromal cells, such as fibroblasts, immune cells, and endothelial cells, to create a tumor-supportive microenvironment [14]. EVs can transfer signaling molecules, growth factors, and cytokines, promoting angiogenesis, extracellular matrix remodeling, and immune suppression [91]. EVs derived from primary tumors can prepare pre-metastatic niches in distant organs, facilitating the colonization of metastatic cells [80]. These EVs can prime the recipient microenvironments by modulating immune responses, inducing angiogenesis, and preparing the extracellular matrix for metastatic cell invasion [87•]. EMT is a crucial process in tumor progression and metastasis. EVs can transfer EMT-inducing factors, including microRNAs, to recipient cells, promoting the acquisition of a mesenchymal phenotype [111]. This phenotypic switch enhances tumor cell invasion, migration, and resistance to therapy [86]. Altogether, these mechanisms could be eventually predicted using EV and EV-cargo analyses in lung cancer patients.

### Lung Cancer Treatment, Resistance, Treatment Response

EVs possess natural characteristics that make them attractive vehicles for therapeutic cargo delivery. They can be engineered to carry anti-cancer agents, such as chemotherapeutic drugs, small interfering RNAs (siRNAs), or immune checkpoint inhibitors [101]. These engineered EVs can selectively target tumor cells, overcome biological barriers, and enhance therapeutic efficacy while minimizing off-target effects [101]. EVs have been implicated in the development of drug resistance in lung cancer, contributing to treatment failure and disease recurrence [112]. They can mediate the transfer of resistance-conferring molecules, such as drug efflux pumps, anti-apoptotic factors, and DNA repair proteins, to recipient cells [113]. This transfer can lead to the acquisition of drug-resistance traits and hinder the efficacy of chemotherapy, targeted therapy, and immunotherapy [114]. EVs themselves or specific components of their cargo can be targeted as therapeutic strategies to overcome drug resistance [115]. Inhibition of EV release, interference with EV uptake by recipient cells, or modulation of EV cargo can sensitize cancer cells to treatment and improve therapeutic outcomes [12].

### Prognostic Value of EVs in Lung Cancer

EVs have shown prognostic value in lung cancer, providing insights into disease progression, metastatic potential, and patient outcomes [116]. Xiang et al. recently published a meta-analysis exploring EVs’ role in lung cancer prognosis. They discovered that the expression level of genes carried by exosomes is closely associated with the poor prognosis of lung cancer. Their results have strong statistical significance, especially for the overall survival and disease-free survival of patients with lung cancer. [117]. Specific EV cargo profiles have been associated with poor prognosis, including increased metastatic potential, resistance to therapy, and shorter overall survival. Certain miRNAs and proteins carried by EVs have been linked to the metastatic potential of lung cancer [118]. For example, EV-miR-203a-3p has been proposed as a relapse biomarker for resected non-small cell lung cancer [118]. Research efforts are needed to evaluate the relevance of EVs in the prognosis of air pollution-related lung cancer. Miguel‐Perez et al. also explore the role of EV PD‐L1 in plasma samples collected before and after immune‐checkpoint inhibitors treatment [119]. They found that increases in EV PD‐L1 were observed in non‐responders in comparison to responders, and EV PD‐L1 resulted in being an independent biomarker for shorter progression‐free survival and overall survival [119]. Rao et al. also explored the role of long noncoding RNAs (lncRNA) in plasma EVs, particularly HAGLR, a lncRNA coded on the antisense strand of a homeobox gene (HOXD) encoding gene on chromosome, which belongs to the HOX family. HAGLR was significantly decreased in NSCLC patients, and they found it associated with overall survival. The high expression of HAGLR was positively correlated with the high detection rate of circulating tumor cells, suggesting the presence of a later tumor stage, which is clearly associated with poor prognosis [120]. These results suggest that EVs may be new biomarkers for the prognosis of lung cancer, although more studies are still needed to be applied in clinical practice.

## Future Avenues

EVs’ research holds significant promise in the field of lung cancer due to their roles in intercellular communication and their potential to serve as sources of valuable information about tumor biology, also contributing as diagnostic and prognostic markers, but also as therapeutic vehicles. Some expected future avenues in EV research related to lung cancer include insisting on the use of EVs and cargo for early detection and diagnosis, which could be helpful in populations living in highly polluted areas. EVs can provide information about phenotypes, stages, and molecular abnormalities, particularly as liquid biopsies, providing insights about the presence and characteristics of the tumors. Studying the cargo within EVs shed by cancer cells will help us understand the molecular mechanisms underlying environmentally related lung cancer progression, invasion, and metastasis. Finally, EV research will help us understand how EVs mediate communication between cancer cells and surrounding cells (e.g., immune cells, fibroblasts, and endothelial cells), leading to strategies to modify the tumor microenvironment for therapeutic benefit.

## Conclusions

Addressing the impact of ambient air pollutants on lung health is highly relevant due to the adverse effects these pollutants have on respiratory symptoms, lung function, and the development of lung cancer [121••]. This risk is more prominent in a climate change context with an increased number of wildfires worldwide [122]. The link between air pollutants and lung cancer is now well-established, with particulate matter, NOx, PM_2.5_, and volatile organic compounds being key contributors [26, 121••]. These pollutants can carry direct carcinogens and induce systemic, long-term inflammation, and oxidative stress in the lung cells, leading to DNA damage, mutations, epigenetic changes, the release of EVs, and the promotion of tumor growth and progression [59, 123]. Inflammatory signaling pathways, sometimes modulated by EVs, facilitate a tumor-promoting microenvironment that supports the development of lung cancer [124•]. Effective strategies and interventions to mitigate the harmful effects of air pollutants, including developing biomonitoring of these interventions, which could include EV and EV-cargo, on lung health are needed [125•]. By addressing air pollution exposures in multiple ways (e.g., promoting clean energy sources, improving industrial practices with stricter emission standards, mandating and incentivizing stricter fuel efficiency standards for vehicles, strengthening air quality standards and regulations, reduce deforestation), we can protect public health and improve outcomes for individuals affected by air pollution-related lung cancer [121••, 126]. EVs are critical components of liquid biopsy that will revolutionize medical follow-up in clinical oncology, especially in lung cancer, analyzing not only their number but their composition (miRNAs, lncRNAs, metabolites, peptides) and understanding tumor phenotypes in plasma without needing access to the tumors directly [127].
